# Respiratory‐related evoked potentials in chronic obstructive pulmonary disease and healthy aging

**DOI:** 10.14814/phy2.15519

**Published:** 2022-12-02

**Authors:** Isabella Epiu, Simon C. Gandevia, Claire L. Boswell‐Ruys, Sophie G. Carter, Harrison T. Finn, David A. T. Nguyen, Jane E. Butler, Anna L. Hudson

**Affiliations:** ^1^ Neuroscience Research Australia Randwick New South Wales Australia; ^2^ University of New South Wales Sydney New South Wales Australia; ^3^ Prince of Wales Hospital Sydney New South Wales Australia; ^4^ College of Medicine and Public Health Flinders University Bedford Park South Australia Australia

**Keywords:** dyspnea, EEG, respiratory sensation

## Abstract

Altered neural processing and increased respiratory sensations have been reported in chronic obstructive pulmonary disease (COPD) as larger respiratory‐related evoked potentials (RREPs), but the effect of healthy‐aging has not been considered adequately. We tested RREPs evoked by brief airway occlusions in 10 participants with moderate‐to‐severe COPD, 11 age‐matched controls (AMC) and 14 young controls (YC), with similar airway occlusion pressure stimuli across groups. Mean age was 76 years for COPD and AMC groups, and 30 years for the YC group. Occlusion intensity and unpleasantness was rated using the modified Borg scale, and anxiety rated using the Hospital Anxiety and Depression Scale. There was no difference in RREP peak amplitudes across groups, except for the N1 peak, which was significantly greater in the YC group than the COPD and AMC groups (*p* = 0.011). The latencies of P1, P2 and P3 occurred later in COPD versus YC (*p* < 0.05). P3 latency occurred later in AMC than YC (*p* = 0.024). COPD and AMC groups had similar Borg ratings for occlusion intensity (3.0 (0.5, 3.5) [Median (IQR)] and 3.0 (3.0, 3.0), respectively; *p* = 0.476) and occlusion unpleasantness (1.3 (0.1, 3.4) and 1.0 (0.75, 2.0), respectively; *p* = 0.702). The COPD group had a higher anxiety score than AMC group (*p* = 0.013). A higher N1 amplitude suggests the YC group had higher cognitive processing of respiratory inputs than the COPD and AMC groups. Both COPD and AMC groups showed delayed neural responses to the airway occlusion, which may indicate impaired processing of respiratory sensory inputs in COPD and healthy aging.

## Graphical abstract

Respiratory‐related potentials (RREPs) evoked by airway occlusion were tested in people with chronic obstructive pulmonary disease (COPD), age‐matched and young controls. Amplitudes of RREP peaks were similar in all participant groups, except for N1 that was larger in young participants. Latencies of RREP components were delayed in COPD and healthy aging compared to young participants. This is the first study to demonstrate that RREP amplitudes do not differ between stable COPD and healthy age‐matched participants.

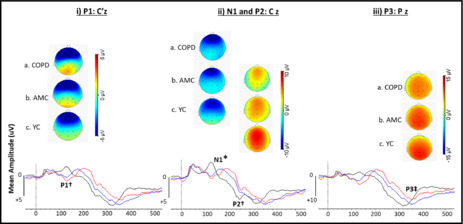

**New and Noteworthy**

1. Respiratory‐related potentials (RREPs) evoked by airway occlusion were tested in people with chronic obstructive pulmonary disease (COPD), age‐matched and young controls.
2. Amplitudes of RREP peaks were similar in all participant groups, except for N1 that was larger in young participants.
3. Latencies of RREP components were delayed in COPD and healthy aging compared to young participants.
4. This is the first study to demonstrate that RREP amplitudes do not differ between stable COPD and healthy age‐matched participants.

## 1 INTRODUCTION

Chronic Obstructive Pulmonary Disease (COPD) is one of the most common non‐communicable diseases, affecting 384 million people worldwide (Global Initiative for Chronic Obstructive Lung Disease (GOLD), 2020). In COPD, airway and alveolar abnormalities result in airflow limitation, increased airway secretions, chronic cough and shortness of breath (dyspnea), a major and debilitating symptom of the disease. Despite comparable clinical presentation (e.g. spirometry) participants experience varying levels of dyspnea (Finnegan et al., 2021). The neural mechanisms that underpin the perception of respiratory sensations in COPD are unclear. In asthma, blunted perception of respiratory loads is linked to poor outcomes (Feldman et al., 2007; Kifle et al., 1997; Magadle et al., 2002), but there is heightened perception of respiratory loads in anxiety, which is very common in COPD (Chan et al., 2012; Livermore et al., 2008; von Leupoldt, Chan, et al., 2011).

Respiratory‐related evoked potentials (RREPs) have long been used to quantify how humans respond to respiratory loads (Davenport et al., 1986, 1992; Eckert et al., 2005; Hudson et al., 2016; Knafelc & Davenport, 1997; O'Donnell et al., 2007). The RREP is an event‐related potential elicited by activation of respiratory mechanoreceptors in the muscles, lungs and airway (Davenport et al., 1986). RREP is analogous to the somatosensory evoked potentials (SEP) elicited by electrical and mechanical stimulation in the limbs (For review see Chan & Davenport, 2010). RREPs are characterized by negative peaks (termed Nf and N1) and positive peaks (P1, P2 and P3) recorded at frontal, central and parietal locations (Chan & Davenport, 2010). Somatosensory activation occurs in the cerebral cortex where the respiratory signals are processed and produce early RREP components, i.e., before ~130 ms (Nf, P1 and N1). The longer latency RREP components (N1, P2, and P3) which come after 150 ms, are related to affective processing and attention and therefore they indicate higher‐order cognitive processing (Chan et al., 2012; Donzel‐Raynaud et al., 2009; Herzog et al., 2018). The N1 component is classified as both an early and late component as it is related to both size of the stimulus (as for P1) and attention (as for P2 and P3; Chan & Davenport, 2010).

Recently, a study of RREPs in participants with COPD revealed higher amplitudes of P1, N1, P2 and P3 when compared to age‐matched control participants, which was interpreted as “greater perception and neural processing of respiratory sensations” in COPD (Reijnders et al., 2020). Although in this study, the mouth pressure change during occlusions was, on average, 73% greater in the COPD group than the age‐matched group (Reijnders et al., 2020). Our first aim was to assess perception and neural processing of respiratory sensations in people with COPD and age‐matched controls with a matched respiratory stimulus between the groups.

Given COPD has been described as accelerated lung aging (Ito & Barnes, 2009; Rutten et al., 2016), the perceptual response to respiratory loads may also be altered in healthy aging. Respiratory mechanics and the neural control of breathing are altered in older adults (Lalley, 2013), including for respiratory muscle reflexes in response to respiratory loads (Epiu et al., 2021). A previous, concise (*n* = 6) study reported differences in RREP peak amplitudes and latencies in older age compared to young participants (Harver et al., 1995). Thus, the second aim of this study was to confirm if healthy aging, in the absence of lung disease, alters the RREP. Therefore, we used electroencephalography (EEG) to assess respiratory‐related cortical activation in people with COPD, age‐matched controls, and young controls.

## 2 METHODS

### 2.1 Ethics

The study procedures were approved by the University of New South Wales Research Ethics Committee (# HC16128). Written informed consent was obtained from each participant. All procedures were conducted in accordance with the Declaration of Helsinki (2013), except for registration in a database (clause 35).

### 2.2 Participants

Thirteen participants with COPD, 14 healthy age‐matched controls (AMC), and 15 healthy young controls (YC) were recruited. People diagnosed with COPD were eligible to participate if they had moderate to severe COPD based on the Global Initiative for Chronic Obstructive Lung Disease (GOLD) criteria (Global Initiative for Chronic Obstructive Lung Disease (GOLD), 2020), i.e. a forced expiratory volume in 1 s (FEV_1_) of <80% predicted, and FEV_1_/forced vital capacity (FVC) ratio (FEV_1_/FVC) of <70%, (Table 1). Healthy AMC and YC participants (18–35 years old) were eligible if they did not have a prior history of asthma, chronic respiratory disease, or neurological diseases.

**TABLE 1:** Anthropometric, spirometry, maximal inspiratory pressure data and anxiety and depression scores

	COPD *n* = 10	AMC *n* = 11	YC *n* = 14	*p*‐value
Age (years)	76 ± 12^a^	76 ± 7^a^	30 ± 6	**<0.001**
Female – N (%)	4 (40)	6 (55)	6 (43)	0.770
BMI kg/m^2^	23.7 ± 2.8^b^	28.5 ± 4.7^a^	24.0 ± 4.4	**0.015**
FEV_1_ (l)^c^	1.2 ± 0.3^a^ ^,^ ^b^	2.1 ± 0.6^a^	3.5 ± 0.7	**<0.001**
FEV_1_% PRED^c^	51 (45, 53)^a^ ^,^ ^b^	88 (77, 113)	87 (80, 91)	**0.004**
FVC (l)^c^	2.4 ± 0.6^a^	2.8 ± 0.8^a^	4.2 ± 0.9	**<0.001**
FVC % PRED^c^	78 ± 16	96 ± 22	87 ± 14	0.151
FEV_1_/FVC (%)^c^	50 ± 9^a^ ^,^ ^b^	76 ± 5	85 ± 8	**<0.001**
PEF (l/s)^c^	157 ± 54^a^ ^,^ ^b^	349 ± 160^a^	457 ± 124	**<0.001**
MIP (cmH_2_O)^d^	62 ± 18^a^	71 ± 35	100 ± 28	**0.021**
HADS‐Depression	1.0 (1.0, 6.0)	2.0 (1.0, 4.0)	—	0.722
HADS‐Anxiety	6.6 ± 4.7	2.0 ± 1.2	—	**0.013**

*Note*: Mean (±SD) or median (IQR) values are shown for participants with chronic obstructive pulmonary disease (COPD), age‐matched controls (AMC) and young controls (YC). ANOVA or Kruskal Wallis tests were performed. *p* Values less than 0.05 are shown in bold.

Abbreviations: % predicted, percent predicted for age, sex, and ethnicity; FEV_1_, forced expiratory volume in 1 s; FVC, forced vital capacity; HADS, Hospital Anxiety and Depression Scale; MIP, maximal inspiratory pressure; PEF, peak expiratory flow.

^a^ Significant post‐hoc difference compared to YC, *p* < 0.05.

^b^ Significant post‐hoc difference compared to AMC, *p* < 0.05.

^c^ *n* = 12 for YC.

^d^ *n* = 8 for YC.

We recruited people with stable COPD who had previously volunteered in our laboratory and agreed to return for future experiments. New participants with COPD were referred from the Prince of Wales Private Hospital based on a diagnosis of COPD. The control participants were recruited from the Neuroscience Research Australia Research Volunteers Registry or were previous volunteers.

### 2.3 Procedure

#### 2.3.1 Spirometry

Forced expiratory lung volumes and forced vital capacity were measured using a hand‐held spirometer (One Flow FVC Memo, Clement Clarke, Harlow, UK or MicroLab, Carefusion), following ATS/ERS guidelines (Miller et al., 2005). At least three attempts of spirometry were performed, until two values were within 10% of each other, and we recorded the highest values (Miller et al., 2005). The predicted FEV_1_ and FVC values were calculated using the European Respiratory Society Global Lung Initiative Calculator (Quanjer et al., 2012). Inspiratory muscle strength was quantified using a MicroRPM Pressure Meter (CareFusion).

#### 2.3.2 Respiratory‐related evoked potentials

Participants were seated comfortably with neck, arm, and leg support in a quiet, soundproof room. EEG was recorded from 12–13 scalp locations according to the internationally recognized 10/20 system as well as A1 and A2 on the earlobes and below the right eye to detect eye blinks (Acticap; BrainProducts, Gilching, Germany; Figure 2). A2 was used as a reference during the recordings and a ground electrode was positioned at AFz. Mouth pressure was recorded as an auxiliary channel in the EEG system. Signals were amplified and filtered at 0.1–500 Hz and sampled at 500 Hz (BrainAmp, Brain Products v. 1.20.0801).

The participant wore a nose‐clip and breathed through a mouthpiece connected to a bacterial and viral filter (SureGard Blue, Bird Healthcare, Victoria, Australia), pneumotachograph (Series 3813; Hans Rudolph), pressure transducer (DP45‐16; Validyne Engineering) and a two‐way valve with a pneumatic balloon valve in the inspiratory port (Series 2600; Hans Rudolph) (for details see Epiu et al., 2021). Airflow was measured continuously and integrated online to record tidal volume. End‐tidal CO_2_ was monitored (Normocap; Datex Instrumentarium, Helsinki, Finland).

Surface electromyography activity (EMG) was recorded from the scalene muscles bilaterally with clear‐trace electrodes (ConMed Corp.; for details see Murray et al., 2008). EMG was amplified (×10,000) and filtered (band pass 16–1000 Hz) with CED 1902 amplifiers (Cambridge Electronic Design Ltd). EMG signals were sampled at 2000 Hz and respiratory signals at 1000 Hz (CED 1401; Spike2, version 7.2; Cambridge Electronic Design) and analyzed offline.

To evoke RREPs, brief airway occlusions of 250 ms were delivered at mid‐inspiration, randomly every 3–5 normal breaths, until at least 50 occlusions were recorded, with 2–3 breaks per session. A custom Spike2 script activated the balloon valve based on a pre‐set volume threshold. Participants were advised to sit still and encouraged to “breathe through” the occlusion as if it was not there, and they received visual feedback of a target flow of 0.5–0.7 L/s. Participants were asked to maintain the target level of flow during their inspiration to increase the chance that the airflow at occlusion, and thus the pressure change, would be equivalent across participants. To block auditory stimuli from the occlusion and other sounds within the room, participants listened to music through noise‐canceling Bluetooth earpieces.

The participants scored the intensity and unpleasantness of the airway occlusions using the modified Borg scale (Borg, 1982; Livermore et al., 2008) posed as separate questions, as used in a recent RREP study in participants with COPD (Reijnders et al., 2020). Anxiety alters respiratory sensations (von Leupoldt, Chan, et al., 2011), therefore anxiety and depression were also assessed with the Hospital Anxiety and Depression Scale (HADS).

### 2.4 Analysis

RREPs were monitored online, and data were saved for offline analysis. The EEG traces were re‐referenced to linked ears (A1‐A2), filtered (0.5–30 Hz), segmented (50 ms before to 450 ms after the onset of the airway occlusion) and inspected for eye blink or movement artifacts. The C'z channel was computed as the average of CP1 and CP2. Traces with big artifacts were omitted from averages, leaving 36–104 occlusions (range) across participants for average waveform data. The mean and median numbers of occlusions were 49 and 50, 57 and 54, and 81 and 87, for participants with COPD, AMC and YC, respectively.

In some participants, EEG signals, especially in the frontal region, picked up an artifact related to the inflation and deflation of the balloon in the breathing circuit during airway occlusions. To determine the potential impact of this artifact, the cross‐correlation coefficients between the average mouth pressure and average EEG signals for Fz, Cz, C'z and Pz were determined for each participant. The mean cross‐correlation coefficients were about double for the COPD and AMC groups than that of the YC group for all EEG channels. They were 0.81, 0.78 and 0.38 for Fz, 0.47, 0.34 and 0.22 for Cz, 0.30, 0.32 and 0.20 for C'z and 0.18, 0.28 and 0.14 for Pz, for COPD, AMC and YC respectively. To account for large negative shifts in EEG signals during balloon inflation, the baseline voltage (i.e., zero) of the EEG channel was set to the average EEG value between 0–250 ms, only for EEG channels with a correlation coefficient between EEG and mouth pressure of *r* > 0.5. A *r* > 0.5 was chosen as it indicated when more than 50% of the variation in the EEG signal between 0–250 ms was due to the deviation in mouth pressure related to inflation of the occlusion balloon, rather than brain‐derived changes in EEG activity. This was the case for 10 COPD, 10 AMC and 7 YC participants for Fz, 3 COPD, 4 AMC and 3 YC for Cz, 3 COPD, 3 AMC and 3 YC for C'z, and 2 COPD, 3 AMC and 1 YC for Pz.

The first positive peak on the centro‐parietal channel C'z was identified as P1, followed by a negative peak N1 and then the second positive peak P2 on the central Cz, and lastly the third positive peak P3 on the parietal Pz channel (Figures 2 and 3 and Table 3, Chan & Davenport, 2010; von Leupoldt et al., 2010). The amplitude and latency of the peaks were measured. We did not analyze data from the Nf peak on Fz channel due to artifact in frontal channels for most participants.

Ventilatory parameters were measured for at least 10 breaths in each participant during quiet breathing (Table 2). From the average waveforms during airway occlusions, the volume and inspiratory flow were measured at the abrupt onset of the negative deflection in mouth pressure. The scalene EMG prior to the occlusion was measured as the mean of the root mean square signal (50 ms time constant) over 100 ms before the occlusion for both the right and left scalenes, and then averaged across participants in each group. The change in negative pressure evoked by airway occlusions was measured from the initial deviation in mouth pressure (i.e., before further volitional increases in mouth pressure over the 250 ms).

**TABLE 2:** Respiratory variables during quiet breathing and airway occlusions

	COPD *n* = 10	AMC *n* = 11	YC *n* = 14	*p*‐value
QB RR (breaths/minute)	20 ± 5^a^	16 ± 5	14 ± 4	**0.007**
QB Tidal volume (l)	0.550 ± 0.084^a^	0.681 ± 0.297	0.791 ± 0.216	**0.042**
QB Minute ventilation (l/m)	10.9 ± 2.56	9.8 ± 3.1	10.8 ± 3.1	0.612
QB Mean flow (l/s)	0.589 ± 0.135	0.537 ± 0.171	0.496 ± 0.104	0.269
QB Inspiratory time (s)	1.2 ± 0.3^a^ ^,^ ^b^	1.7 ± 0.6	1.7 ± 0.4	**0.025**
QB Pm (cmH_2_O)	−1.19 ± 0.22^a^ ^,^ ^b^	−1.24 ± 0.25	−0.94 ± 0.21	**0.0048**
ET CO_2_ (%)	3.5 ± 1.4^a^ ^,^ ^b^	4.5 ± 0.6	4.6 ± 0.4	**0.006**
Pre‐occlusion EMG Sca (μV)	17.8 (12.5, 25.0)^b^	8.7 (7.9, 12.7)	12.5 (8.0,15.6)	**0.042**
Volume at Occlusion (l)	0.282 ± 0.076	0.278 ± 0.098	0.347 ± 0.097	0.126
Flow at Occlusion (l/s)	0.713 ± 0.075	0.626 ± 0.146	0.649 ± 0.145	0.294
Occlusion Δ Pm (cmH_2_O)	4.1 (3.5, 4.5)	3.5 (2.7, 3.9)	3.4 (2.5, 3.9)	0.089
Occlusion Intensity^c^	3.0 (0.5, 3.5)	3.0 (3.0, 3.0)	—	0.476
Occlusion Unpleasantness^c^	1.3 (0.1, 3.4)	1.0 (0.75, 2.0)	—	0.702

*Note*: Mean (±SD) or median (IQR) values are shown for participants with chronic obstructive pulmonary disease (COPD), age‐matched controls (AMC) and young controls (YC). ANOVA or Kruskal Wallis tests were performed. *p* Values less than 0.05 are shown in bold.

Abbreviations: ET CO_2_, end tidal carbon dioxide; Pm, mouth pressure; QB, quiet breathing; RR, respiratory rate; Sca, Scalene muscles (average of right and left scalene muscles).

^a^ Significant post‐hoc difference compared to YC (Young Controls): *p* < 0.05.

^b^ Significant post‐hoc difference compared to AMC (Age‐matched Controls): *p* < 0.05.

^c^ *n* = 9 for COPD and AMC, *n* = 0 for YC.

All data were compared using a one‐way ANOVA or the Kruskal Wallis test if the data did not pass Shapiro's test of normality. Pairwise post‐hoc tests (Tukey's or Dunn's multiple comparison tests) were also performed to see which of the three groups differed. Pearson correlations (or Spearman's rank correlations when data were non‐parametric) were performed to check for linear associations between Borg and HADS‐Anxiety scores with RREP amplitude. Data are expressed as mean ± SD or median (interquartile range). Statistical significance was set at *p* < 0.05.

## 3 RESULTS

### 3.1 Participants

**FIGURE 1:**
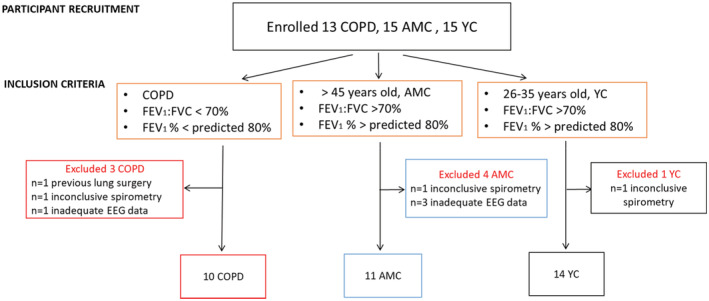
Flow chart of participants recruitment. Participants had moderate to severe chronic obstruction pulmonary disease (COPD), were age‐matched controls (AMC) or young controls (YC). Some enrolled participants were excluded from analysis as they did not meet inclusion criteria or had incomplete data. Abbreviations: FEV_1_: forced expiratory volume in 1 s; FVC: forced vital capacity; % predicted: percent predicted for age, sex, and ethnicity; and EEG: electroencephalography.

Of the participants recruited, three with COPD were excluded due to previous lung surgery, inconclusive spirometry, or incomplete data (Figure 1). One AMC and one YC were also excluded due to inconsistent spirometry and three participants in the AMC group had incomplete data (Figure 1).

Table 1 summarizes the anthropometric and spirometry data. The mean age was 76 ± 12 years in the COPD group, 76 ± 7 years in the AMC group, and 30 ± 6 years in the YC group (*p* < 0.001). The BMI was significantly lower in the COPD group (23.7 ± 2.8 kg/m^2^) compared to the AMC group (28.5 ± 4.7 kg/m^2^) but not the YC group (24.0 ± 4.4 kg/m^2^; *p* = 0.001). As expected, the spirometry values differed between groups with a significantly lower FEV_1_% predicted of 51 (45, 53) % predicted and FEV_1_/FVC ratio of 50 ± 9% for the COPD group, compared to AMC and YC groups (see Table 1). The HADS‐Anxiety scores were higher in the COPD than AMC group (*p* = 0.013).

### 3.2 Respiratory parameters during quiet breathing and inspiratory occlusions

During quiet breathing, all groups had similar ventilation, but respiratory frequency was highest in the COPD group (Table 2). During sudden airway occlusions, the mouth pressure changes were similar for the COPD 4.1 (3.5, 4.5) cmH_2_O, AMC 3.5 (2.7, 3.9) cmH_2_O, and YC 3.4 (2.5, 3.9) cmH_2_O groups (*p* = 0.089; Table 2). Both COPD and AMC participants rated the intensity and unpleasantness of the occlusions similarly with the Borg score (Table 2). The median un‐normalized pre‐occlusion scalene EMG was significantly higher in the COPD group at 17.8 (12.5, 25.0) μV, compared to the AMC group at 8.7 (7.9, 12.7) μV (*p* = 0.042; Table 2), reflecting a higher neural respiratory drive due to their COPD (De Troyer et al., 1997; Gandevia et al., 1996; Jolley et al., 2009).

### 3.3 RREP amplitudes and latencies

**FIGURE 2:**
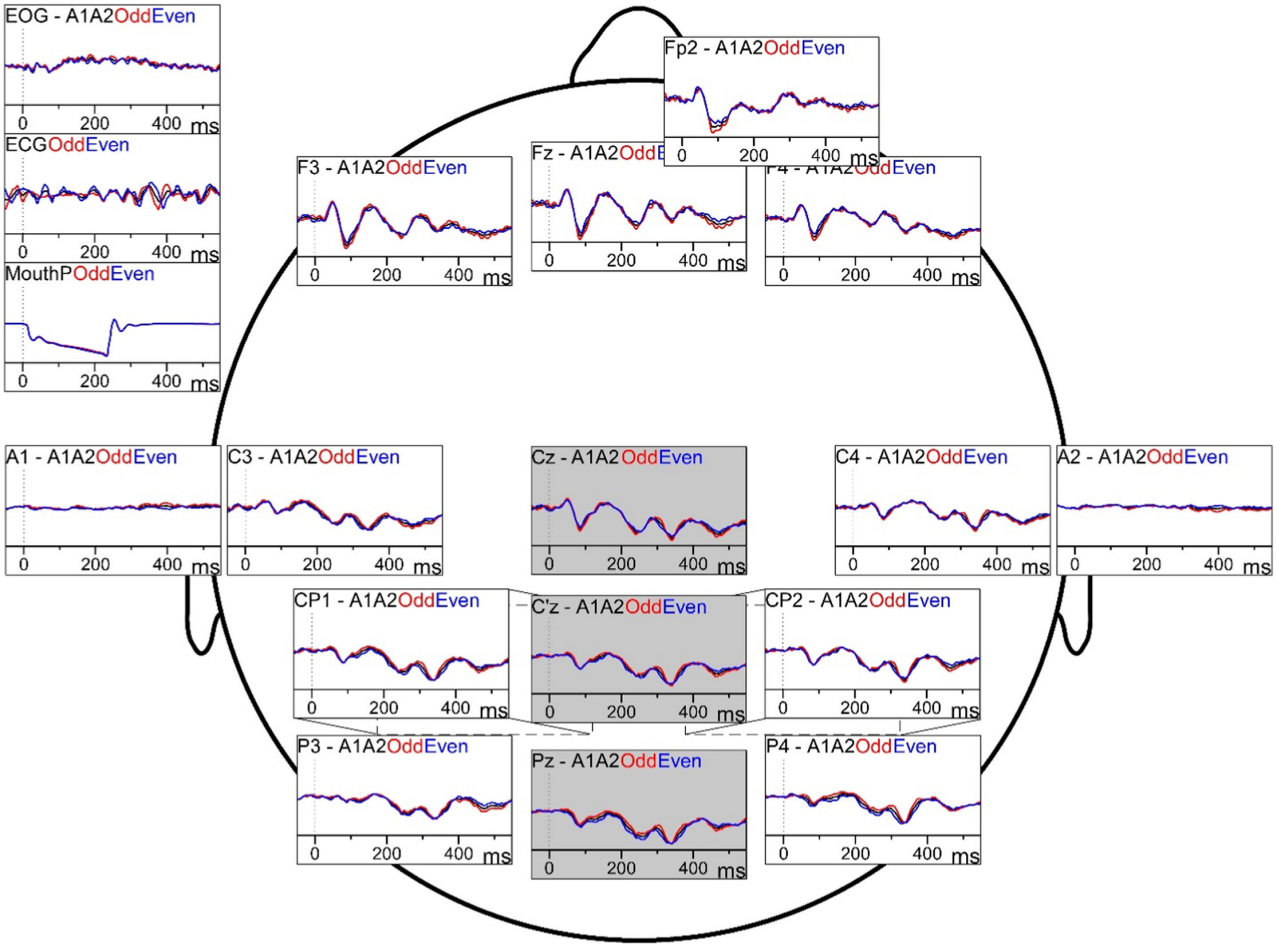
Respiratory‐related evoked potential (RREP) waveforms from one individual on all scalp locations. Average waveforms of the RREP for all (black), odd (red) and even (blue) trials of EEG in a young control participant. EEG was recorded from 12 frontal, central, central parietal, and parietal locations (FP1 was also recorded in some participants), as well as A1 and A2 on the earlobes and below the right eye to detect eye blinks (EOG). Mouth pressure (MouthP) and ECG were recorded as auxiliary channels and EEG trials were time‐locked to the onset of negative inspiratory pressure evoked by brief airway occlusions. All channels were referenced to linked ears, i.e. A1 and A2, and the amplitude and latencies of the RREP peaks were measured from the Cz, C'z and Pz channels (shaded panels, see Methods). C'z channel was computed as the average of CP1 and CP2 as the C'z location was not available on the EEG cap. See Table 3 for RREP group averages.

**FIGURE 3:**
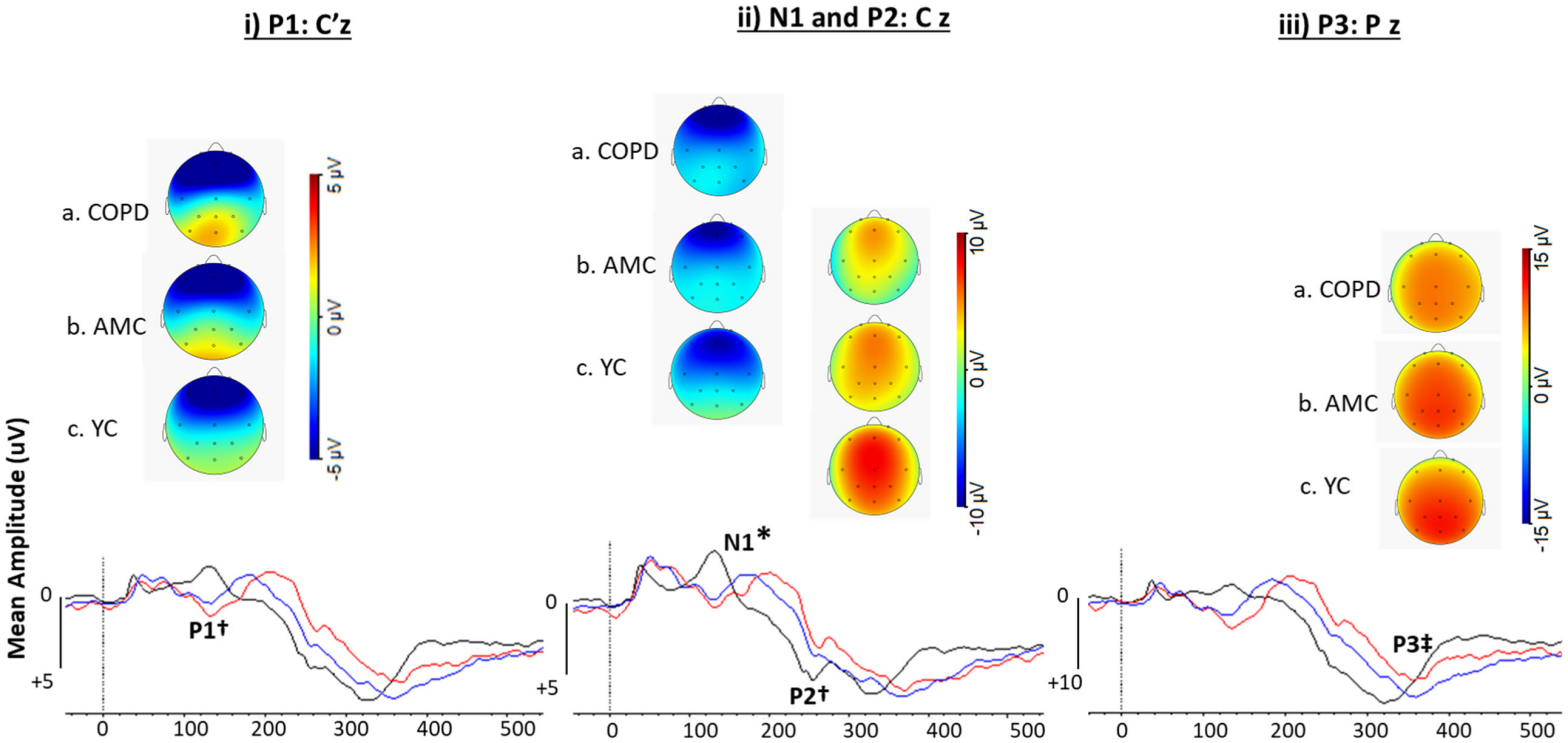
Average respiratory‐related evoked potentials (RREP) and topographic maps for P1, N1, P2, and P3 peaks. Grand average waveforms across all participants of RREPs at (i) C'z, (ii) Cz, and (iii) Pz in chronic obstructive pulmonary disease (COPD; red line), age‐matched controls (AMC; blue line) and young controls (YC; black line). Topographic maps show 2‐dimensional view of EEG across all channels for 20 ms around each peak in the grand average for each group. The peaks are indicated on the EEG channels from which they were measured. †Significant difference in P1 and P2 latency between YC and COPD groups. *Significant difference in N1 amplitude between YC and both COPD and AMC groups. ‡Significant difference in P3 latency between groups (see Table 3).

Respiratory‐related evoked potentials following sudden airway occlusion were observed in the COPD, AMC, and the YC groups. The RREP peaks were measured at C'z (P1), Cz (N1), Cz (P2) and Pz (P3) (see Figures 2 and 3). After adjusting the baseline voltage over 0‐250 ms to account for pressure‐related signal artifact (for EEG channels with a high cross‐correlation with mouth pressure, *r* > 0.5; see Methods), positive P1, P2 and P3 peaks were observed in all subjects (*n* = 35) except for 1 COPD, 2 AMC and 4 YC participants where the P1 peak was measured as a negative voltage. The same COPD participant also had a negative P2 peak. A different COPD participant had a positive voltage N1 peak. All peaks were included in the average amplitudes and latencies.

The RREP peak amplitudes were similar across all groups, except for N1 which was larger in the YC compared to both COPD and AMC groups (Table 3). The latencies of the RREP peaks did differ between participant groups (Table 3), with longer latencies in COPD compared to the YC group for P1, P2 and P3 peaks. However, the latencies in AMC did not differ from the YC group, except for P3. The latencies of the N1 peaks were similar across groups (Table 3).

**TABLE 3:** Amplitudes and latencies of respiratory‐related evoked potential (RREP) peaks

	COPD *n* = 10	AMC *n* = 11	YC *n* = 14	*p*‐value
A. Amplitude (μV)				
P1, C'z	2.90 ± 2.01	2.54 ± 1.87	1.53 ± 2.60	0.724
N1, Cz	−3.48 ± 1.93^a^	−3.43 ± 1.57^a^	−6.34 ± 3.44	**0.011**
P2, Cz	6.56 ± 5.77	8.46 ± 6.17	9.57 ± 5.09	0.444
P3, Pz	8.38 (7.35, 12.53)	11.61 (6.11, 12.40)	13.29 (11.39, 15.96)	0.066
B. Latency (ms)				
P1, C'z	115 (70, 142)^a^	88 (60, 130)	60 (52, 74)	**0.007**
N1, Cz	178 ± 52	153 ± 50	146 ± 30	0.206
P2, Cz	273 (235, 302)^a^	276 (252, 296)	242 (189, 254)	**0.023**
P3, Pz	359 (332,377)	360 (314, 388)	332 (301, 341)	**0.024**

*Note*: Mean (±SD) or median (IQR) amplitudes and latencies of RREP peaks in participants with chronic obstructive pulmonary disease (COPD), age‐matched controls (AMC) and young controls (YC) are shown. The EEG channel on which the peak was measured is indicated. ANOVA or Kruskal Wallis tests were performed. *p* Values less than 0.05 are shown in bold.

^a^ Significant difference compared to YC, *p* < 0.05.

### 3.4 Correlations between RREP amplitude with HADS‐Anxiety and Borg scores

There was no association between P3 amplitude and the HADS‐Anxiety score for the COPD group (*r* = 0.380, *p* = 0.312), or the AMC group (*r* = 0.08 and *p* = 0.843). Additionally, no correlations were observed between the P3 amplitude and Borg score intensity (*r* = −0.162, *p* = 0.680, and *r* = −0.412 *p* = 0.444); or unpleasantness (*r* = −0.181, *p* = 0.669, and *r* = −0.442, *p* = 0.237); for the COPD or AMC groups, respectively.

## 4 DISCUSSION

RREP peaks in response to airway occlusion occurred in the COPD, age‐matched controls (AMC), and the young control (YC) groups as measured at the central, central‐parietal, and parietal regions. There was no evidence of a difference in the RREP peak amplitudes and latencies between the COPD and AMC groups, but some differences emerged when compared to the YC group. The N1 peak amplitude was significantly larger (by 82%) in the YC group than the COPD and AMC groups, and the latencies of some RREP components were prolonged in the older participants, typically those from the COPD group.

### 4.1 Characteristics of the RREP

#### 4.1.1 P1

The early RREP peaks are exclusively related to respiratory sensory perception, i.e., detection and magnitude estimation of sensory input, as the amplitudes of Nf and P1 peaks correlate with the stimulus magnitude, but not with attention, nor emotion (Chan & Davenport, 2010; von Leupoldt et al., 2013; von Leupoldt, Chan, et al., 2011). Here, P1 amplitude was comparable between COPD, AMC and YC groups when similar changes in mouth pressure were evoked during airway occlusion. Previously, P1 amplitude has been reported to be larger in COPD than in age‐matched controls during airway occlusion (Reijnders et al., 2020). However, in that study this may well have occurred because the change in mouth pressure was also much greater in the COPD group, and within that group, P1 amplitude correlated with the change in mouth pressure (Reijnders et al., 2020). However, it should also be considered that it may be due to differences in the participant cohorts (e.g., higher levels of anxiety and perceived unpleasantness of the stimulus in the previously published study, Reijnders et al., 2020) or the methodology (see Limitations below). Of relevance, P1 amplitude and latency are also similar for non‐asthmatic and asthmatic children (Davenport et al., 2000). However, P1 is absent in 55% of children with life‐threatening asthma (Davenport et al., 2000), suggesting that some children with life‐threatening asthma have impaired perception and neural processing of the sensory input associated with the airway occlusion (Davenport et al., 2000).

#### 4.1.2 N1

Unlike the P1 component of the RREP, N1, with a latency of more than 150 ms, can be altered by affective factors, analogous to later components of somatosensory evoked potentials, in which the latency decreases and amplitude increases with attention (Chiappa, 1990; Webster & Colrain, 2000). In addition, N1 represents RREP ‘gating’, a process by which redundant respiratory‐related sensory information is filtered from higher order central processing (Davenport & Vovk, 2009; Gora et al., 2002; Herzog et al., 2018). Here, N1 was 82% larger in the YC than the COPD and AMC groups. This is consistent with a previous study that could not identify the N1 component in some older participants (*n* = 6, mean age 61.5 years) compared to younger participants (*n* = 6, mean age 26.5 years; Harver et al., 1995). N1 amplitude during airway occlusion has previously been shown to vary with the level of background dyspnea, with the greatest N1 amplitude during a no dyspnea condition in young healthy controls (Herzog et al., 2018). In our study, while the COPD and AMC groups rated the intensity and unpleasantness of the occlusions to be similar, unfortunately we did not ask the YC group to rate these sensations nor did we not ask any participant group about their background level of dyspnea. Thus, the larger N1 amplitude in the YC group may be due to greater attentiveness to the stimuli or lower levels of dyspnea during quiet breathing and/or during airway occlusions. Alternatively, the larger N1 amplitude in the YC may indicate a greater ability in this group to ‘gate’ redundant respiratory sensations, which in turn may be linked to their expected lower levels of dyspnea (For review see Gora et al., 2002). Consistent with our findings, N1 amplitude is also reduced in obstructive sleep apnea (OSA) compared to control participants (in whom the EEG was also referenced to linked ears (A1‐A2); Gora et al., 2002).

People with OSA, COPD and aging generally have impaired sensory neural function compared to healthy young control participants. For example, inspiratory load perception is increased in COPD, but only in those with panic attacks or panic disorders (Livermore et al., 2008), and load perception is reduced in OSA (Tun et al., 2000). Load detection, on the other hand, is blunted in OSA and aging (Altose et al., 1985; McNicholas et al., 1984; Ruehland et al., 2017), which may also contribute to a reduced N1 amplitude and delayed N1 latency in these groups. Of note, the inspiratory muscle inhibitory reflex responses evoked by airway occlusions are also comparably prolonged in COPD, AMC and OSA (Epiu et al., 2021; Jeffery et al., 2006), suggesting an impairment in respiratory sensory processing common across these groups.

#### 4.1.3 P2 and P3

In the current study, the amplitudes of the later P2 and P3 components were similar across the groups. We saw no correlation between the measures of intensity and unpleasantness with P3 amplitude within the COPD or AMC groups. However, in the study by Reijnders and colleagues (Reijnders et al., 2020), the COPD group rated the occlusion intensity and unpleasantness as higher than the AMC, and for the COPD group, P3 amplitude correlated with ratings of both the intensity and unpleasantness of the airway occlusions (Reijnders et al., 2020). A greater amplitude of the P3 peak can reflect increased cognitive processing and greater attention to the stimulus (von Leupoldt, Chan, et al., 2011). In our study, the COPD and AMC groups reported similar ratings of the intensity and unpleasantness of the occlusion, consistent with the observed similar P2 and P3 amplitudes across groups. However, our occlusion lasted only 250 ms (cf. 600 ms Reijnders et al., 2020) and airflow was targeted at 0.5–0.7 L/s in an attempt to deliver a consistent negative pressure stimulus across groups (cf. Reijnders et al., 2020 where COPD group experienced a larger stimulus). The longer duration of the occlusion (2.4 times longer) could have exaggerated the intensity or unpleasantness scores in the previous study (Reijnders et al., 2020). These factors combined with the higher anxiety scores (Livermore et al., 2008; see below), may contribute to the differences in the findings.

Epidemiological studies have reported that anxiety and depression affect 21%–96% and 27%–79% of COPD patients, respectively (Gordon et al., 2019; Yohannes et al., 2000). Perceived unpleasantness of respiratory loads is greater for people with COPD with high levels of anxiety (Livermore et al., 2008), and this can be normalized after a short treatment with tailored cognitive behavioral therapy (Livermore et al., 2015). The HADS‐Anxiety score for the COPD group in Reijnders and colleagues' study (Reijnders et al., 2020), was 7.6 ± 3.1 (mean ± SD), defined as ‘borderline abnormal’, but no HADS‐Anxiety score is available for their controls. A HADS‐Anxiety score of 0–7 (from a maximal score of 21) is considered normal. Increased anxiety traits may be linked to higher amplitudes of P2 and P3 (von Leupoldt, Chan, et al., 2011). However, in the current study, while our COPD group had higher HADS‐Anxiety scores compared to the AMC group (6.6 ± 4.7 versus 2.0 ± 1.2, respectively), only two individuals with COPD had HADS‐Anxiety scores >7. Thus, in our study, in contrast to previous data (Reijnders et al., 2020), anxiety may not have modulated the P2 and P3 responses in the COPD and AMC groups as the mean HADS‐Anxiety scores were within the normal range.

#### 4.1.4 Latencies

The latencies of the P1, P2 and P3 were longer in the COPD group than the YC group, by about 55 ms, 30 ms and 25 ms, respectively. The P3 latency was also longer in AMC than in the YC group by ~30 ms. Our findings are consistent with previous reports of longer latencies of RREPs in older participants with a mean age of 61.5 years (Harver et al., 1995), and individuals with OSA (Kotterba et al., 1998; Sangal & Sangal, 1997). These delays may indicate an age‐related and/or disease‐related sensory impairment in this group resulting in a reduced neural response to the airway occlusion (Davenport et al., 1986; Donzel‐Raynaud et al., 2004; Knafelc & Davenport, 1997). Additionally, nerve conduction velocity also slows with aging (Baudry et al., 2015), which results in more dispersed afferent volleys which could then delay and reduce the amplitude of peaks in the RREP (Buchthal & Rosenfalck, 1966). Without a control task in our study, we cannot determine if longer RREP latencies reflect a generalized age‐related decline in somatosensory processing or if they are specific to the respiratory system. Future studies could address this using both respiratory‐related evoked potentials and potentials evoked in a non‐respiratory task (e.g. auditory or visual).

### 4.2 Limitations

Several studies have documented neural and respiratory impairments in aging (Cosio et al., 2014; Fjell et al., 2014; Lalley, 2013; Monk et al., 2008; Navaratnarajah & Jackson, 2013). In our study, the mean age of the COPD and AMC groups was more than double (2.5 times) that of the YC group, with significant differences in lung function and inspiratory muscle strength. A difference in muscle strength between groups is unlikely to explain any differences, or lack of differences, in the RREP, as inspiratory muscle training that increases occlusion mouth pressure at 0.1 s and maximal inspiratory pressure does not alter the RREP (Huang et al., 2003). The COPD group, in our study, had a greater level of pre‐occlusion EMG compared to AMC, which fits with their higher respiratory neural drive required for resting breathing (De Troyer et al., 1997; Gandevia et al., 1996; Jolley et al., 2009). Due to the greater inspiratory muscle activity in participants with COPD, it is possible active muscle stiffness may be higher in this group. Thus, because the muscles are potentially shortening at a higher rate and may be stiffer, a similar change in inspiratory load (assessed by change in mouth pressure) might act as a larger stimulus. Despite this, the amplitudes or latencies of the RREPs were similar in the COPD and AMC groups.

In the current study, we referenced the EEG to linked ears (A1‐A2), which resulted in RREPs with amplitudes and latencies comparable to those published previously using the same reference (e.g. Eckert et al., 2005 [personal communication]). We were unable to reference to an average of all scalp locations as used previously to measure RREPs in COPD (Reijnders et al., 2020) as we had a low density of EEG recordings (12 or 13 locations), and artifact on the frontal channels. The observed mouth pressure‐related EEG artifacts in the frontal channels in several participants meant that we were unable to analyze the Nf data on Fz reliably, but for the other EEG channels we performed a customized baseline correction to measure other RREP peaks. As the cross‐correlations between EEG and mouth pressure were comparable in the COPD and AMC groups, as well as the proportion of participants from both groups whose baseline EEG was corrected, we do not believe the lack of difference in RREP peak amplitudes between these groups was associated with the artifact or subsequent artifact corrections.

The number of occlusions used to evoke RREP with our methodology was adequate (Revelette & Davenport, 1990, see also von Leupoldt, Keil, et al., 2011), but we had a small number of participants in the COPD and AMC groups. In the current study, P1 amplitude in the COPD group was not different from the AMC or YC groups (Table 3), perhaps due to the variation within the groups. Thus, although the inability to reproduce the previous RREP results of Reijnders and colleagues (Reijnders et al., 2020) may be explained by the matched changes in mouth pressure during airway occlusions across COPD and control groups in the current study, a larger study should be done to confirm this.

## 5 CONCLUSION

This is the first study to demonstrate that RREP amplitudes do not differ between stable COPD and healthy age‐matched participants. However, compared to these elderly participants, healthy young controls had a larger N1 peak. The latencies of the RREP components were longer in the elderly participants, particularly in COPD. This study confirms altered neural responses to airway triggers and the neural control of breathing in healthy aging, in participants with and without COPD. More research to assess neural processing of respiratory sensations in acute exacerbations of COPD is recommended. Given the levels of anxiety in people with COPD (Livermore et al., 2010), it would be helpful to examine the effect of psychotherapeutic interventions on RREPs with the aim to improve clinical outcomes, and the quality of life in people with chronic pulmonary diseases.

## AUTHOR CONTRIBUTIONS

All authors contributed to the study conception. I.E., D.A.T.N, S.G.C., H.T.F, C.L.B‐R. and A.L.H performed the experiments at Neuroscience Research Australia. I.E., and A.L.H. analyzed the data. I.E. wrote the manuscript. I.E., S.C.G., J.E.B., and A.L.H. edited and revised the manuscript. All authors interpreted the data and revised the manuscript. All authors approved the final version of the manuscript.

## FUNDING INFORMATION

This work was supported by the National Health and Medical Research Council (NHMRC Australia ‐ 1138920) and the Rebecca L. Cooper Medical Research Foundation. I.E., is funded by a University of New South Wales, Scientia PhD Scholarship, S.C.G. and J.E.B. are supported by NHMRC Fellowships.

## DISCLOSURE STATEMENTS

Financial disclosure: none. Non‐financial disclosure: none.
